# Advances of Cell Membrane-Coated Nanotechnology and Membrane Vesicles in Intestinal Targeted Drug Delivery Systems

**DOI:** 10.3390/pharmaceutics18050534

**Published:** 2026-04-27

**Authors:** Rou Tang, Fujun Zeng, Chengzhen Lyu, Jianyekai Tuerheng, Ziqi Guo, Kun He, Dong Wu

**Affiliations:** 1 Department of Pharmacy, Peking Union Medical College Hospital, Chinese Academy of Medical Sciences & Peking Union Medical College, Beijing 100730, China; tangrou@pumch.cn; 2 Department of Gastroenterology, State Key Laboratory of Complex Severe and Rare Diseases, Peking Union Medical College Hospital, Chinese Academy of Medical Sciences & Peking Union Medical College, Beijing 100730, China; 3 Beijing Key Laboratory of Drug Delivery Technology and Novel Formulation, Institute of Materia Medica, Chinese Academy of Medical Sciences & Peking Union Medical College, Beijing 100050, China; 4 Department of Gastroenterology, People’s Hospital of Xizang Autonomous Region, Lhasa 850000, China

**Keywords:** cell membrane-coated nanotechnology, cell membrane-coated nanoparticles, intestinal drug delivery, biomimetic nanocarriers, membrane vesicles

## Abstract

Although nanomedicine has enabled significant advances in drug delivery, the clinical translation of conventional synthetic nanocarriers is limited by immune clearance, non-specific biodistribution, and gastrointestinal instability. This poses major challenges for therapy targeting the intestines. Cell membrane-coated nanotechnology (CMCT) and membrane vesicle-based systems have emerged as biomimetic platforms integrating synthetic nanomaterials with naturally derived biological interfaces. These biohybrid systems inherit biological functions originating from cells, including immune evasion, prolonged circulation, lesion homing, and microenvironment-responsive interactions, through the direct transfer of intact membrane components. This review summarizes recent advances in CMCT and membrane vesicle-based strategies for intestinal drug delivery. It covers fabrication methodologies, programmable manufacturing approaches, and functional regulation enabled by diverse membrane sources and hybrid engineering designs. Applications in inflammatory bowel disease, colorectal cancer, and intestinal infections are highlighted, emphasizing key therapeutic mechanisms, such as targeting inflammation, neutralizing toxins, modulating the immune system, and regulating the microbiome. We also discuss the major challenges of translation, such as preserving membrane and coating integrity, ensuring oral stability, achieving batch reproducibility, and ensuring biosafety. Overall, this review establishes a conceptual and engineering framework to guide the transition of membrane-based nanocarriers from passive biomimicry to adaptive, clinically translatable intestinal delivery systems.

## 1. Introduction

Advancements in nanotechnology and materials science have driven the development of diverse nanomedicines, including liposomes, micelles, and nanoparticles, some of which have already entered clinical use [1,2,3]. Among these platforms, liposomes are the most clinically established drug delivery systems (DDS), with over a dozen formulations approved for clinical use [4,5,6]. These systems can improve therapeutic efficacy, reduce off-target effects, and support site-specific drug delivery. However, the broader clinical translation of conventional nanocarriers remains limited by several well-recognized barriers. One major limitation is insufficient biocompatibility and the risk of carrier-related toxicity. Certain inorganic or synthetic polymeric nanocarriers can induce oxidative stress, inflammation, and cellular damage, thereby compromising both safety and therapeutic performance [7,8,9]. In addition, nonspecific biodistribution can cause drug accumulation in healthy tissues, reducing therapeutic efficacy and increasing systemic toxicity [10]. Rapid immune clearance by the mononuclear phagocyte system (MPS) further shortens circulation half-life and limits accumulation at target sites [11,12]. These limitations are even more pronounced in oral and intestinal delivery, where gastric acidity, digestive enzymes, the mucus layer, and epithelial tight junctions collectively restrict nanoparticle stability, transport, and absorption, resulting in low bioavailability and short-lived therapeutic exposure [13,14,15]. These route-specific barriers make intestinal drug delivery fundamentally different from systemic nanomedicine design and create a need for delivery platforms with improved biological adaptability.

In response to these challenges, biomimetic delivery strategies have attracted increasing attention because they are better suited to interact with complex physiological environments. Cell membrane coating nanotechnology (CMCT) has emerged as one such strategy. Conventional bottom-up functionalization strategies can prolong circulation or provide ligand-based specificity, but they do not fully reproduce the complexity of native biological interfaces [16,17,18]. In contrast, CMCT is a top-down approach in which natural cell membranes are transferred onto synthetic nanoparticle cores. This design preserves membrane-associated components from source cells and produces cell membrane-coated nanoparticles (CMNPs) with biologically active surface interfaces [16,19]. These biohybrid systems combine the engineering flexibility of synthetic cores with the biological functions of natural membranes. Compared with conventional modification such as polyethylene glycol (PEG) functionalization or ligand conjugation, CMCT can provide better biocompatibility, immune compatibility, and functional diversity [20,21,22]. CMCT therefore offers a more complete biological interface for nanoparticle design and targeted delivery.

Functionally, CMNPs retain selected biological features of their source membranes, which may support immune evasion, lesion recognition, or microenvironmental interaction. For example, membranes enriched with self-recognition proteins such as cluster of differentiation 47 (CD47) and complement regulatory proteins can reduce MPS-mediated clearance and prolong circulation [23,24]. Immune- or platelet-derived membranes may also enhance localization to inflamed or injured tissues through naturally evolved adhesion and homing properties [25,26,27,28]. In addition, CMCT is readily adaptable through membrane engineering and hybridization, which expands its potential across different disease settings [29,30,31,32]. These features have made CMCT-based nanoplatforms attractive for drug delivery and related biomedical applications.

CMCT has therefore become an important direction in biomimetic drug delivery system design. Its versatility allows functional tuning through membrane source selection, hybridization, and molecular engineering, facilitating multifunctional biomedical applications. For intestinal drug delivery in particular, this approach is of interest because it may help address both general nanocarrier limitations and route-specific barriers in the gastrointestinal tract. In this review, we summarize recent advances in CMCT, with a focus on membrane sources, functional design, and therapeutic applications in intestinal diseases. We first outline the conceptual basis and design features of CMCT, then discuss major membrane sources and their functional characteristics, followed by recent applications in intestinal diseases and the main barriers to clinical translation. We also discuss the main challenges to translation, including manufacturing reproducibility, membrane stability, immunogenicity, and oral delivery robustness.

## 2. CMCT: Concept, Fabrication, and Current Limitations

Over the past few decades, synthetic surface functionalization strategies have been widely investigated to improve the biological performance of nanoparticles. These bottom-up approaches, based on the controlled assembly of polymers or targeting ligands, may emulate selected biological interactions, but they do not fully capture the complexity of native cell surfaces. It is now widely recognized that synthetic materials, being foreign to biological environments, are often rapidly identified and eliminated by immune surveillance mechanisms. This limitation has driven increasing interest in biomimetic design strategies inspired by naturally evolved biological structures and processes. By leveraging evolutionarily optimized mechanisms of self-recognition, immune regulation, and intercellular communication, biomimetic nanotechnology provides a framework that integrates synthetic controllability with biological functionality, enabling nanoparticles to function effectively within physiological environments [33,34,35,36]. Within this broader framework, CMCT has emerged as a representative strategy because it transfers intact membrane components onto synthetic cores rather than attempting to recreate isolated functions through chemical modification.

### 2.1. What CMCT Is and Why It Differs from Conventional Surface Engineering

Cell membranes function as the key interfaces between cells and their surrounding biological environment. Their relevance to CMCT lies mainly in the preservation of membrane-associated proteins, glycans, and other surface components involved in recognition, adhesion, signaling, and immune regulation [37,38,39,40]. Because these features differ among cell types, membrane source selection can strongly influence the biological behavior of the resulting nanocarriers. Biomimetic delivery strategies have therefore been developed to reproduce selected biological functions in synthetic systems, either by mimicking individual cellular features or by directly transferring natural membrane components [41,42]. Compared with strategies that replicate only isolated aspects of cell behavior, CMCT is distinctive in that it reconstructs a more biologically relevant surface interface through direct membrane transfer.

Among these approaches, coating nanoparticle cores with natural cell membranes represents one of the most direct ways to reconstruct biologically relevant interfaces on synthetic nanomaterials [43]. In this top-down strategy, preformed nanoparticle cores are combined with natural cell membranes to generate biohybrid systems that preserve the physicochemical advantages of synthetic materials while retaining selected surface functions of the donor cell. As a result, the behavior of CMNPs depends strongly on membrane source and may be tuned for circulation support, targeting, or immune interaction. Compared with conventional chemical surface modification, this strategy provides a more biologically relevant interface and can improve biocompatibility, functional adaptability, and targeting performance [16].

A pioneering study published in 2011 established the feasibility of CMCT by showing that entire cellular membranes could be used to functionalize synthetic nanoparticles [23]. In that study, red blood cell (RBC) membranes were transferred onto poly(lactic-co-glycolic acid) (PLGA) nanoparticle cores to form core–shell structures that preserved key membrane components. RBC membrane-coated nanoparticles showed markedly prolonged circulation half-life compared with PEGylated nanoparticles, largely because self-recognition markers such as CD47 and complement regulatory proteins were retained [44,45]. This work demonstrated that biologically functional interfaces could be introduced by membrane transfer rather than by stepwise chemical decoration. Building upon this foundation, Zhang and colleagues reported the first platelet membrane–coated nanoparticles (PNPs) in 2015 [46]. Platelet membranes enabled nanoparticles to retain vascular injury recognition, immune evasion, and pathogen-binding properties through preserved adhesion receptors and immunomodulatory molecules [47,48,49]. This study further illustrated that CMCT could preserve multiple native membrane functions simultaneously on a synthetic core. These platelet-coated particles displayed affinity for damaged vasculature and microbial targets while maintaining immune-compatible surface features [49]. In relevant disease models, such systems improved delivery efficiency and therapeutic performance, highlighting the translational potential of CMCT. Subsequent studies expanded CMCT to membranes from many different cell types, establishing a broader technological basis for multifunctional therapeutic and diagnostic nanoplatforms [50,51].

### 2.2. How CMCT Is Fabricated

**Figure 1:**
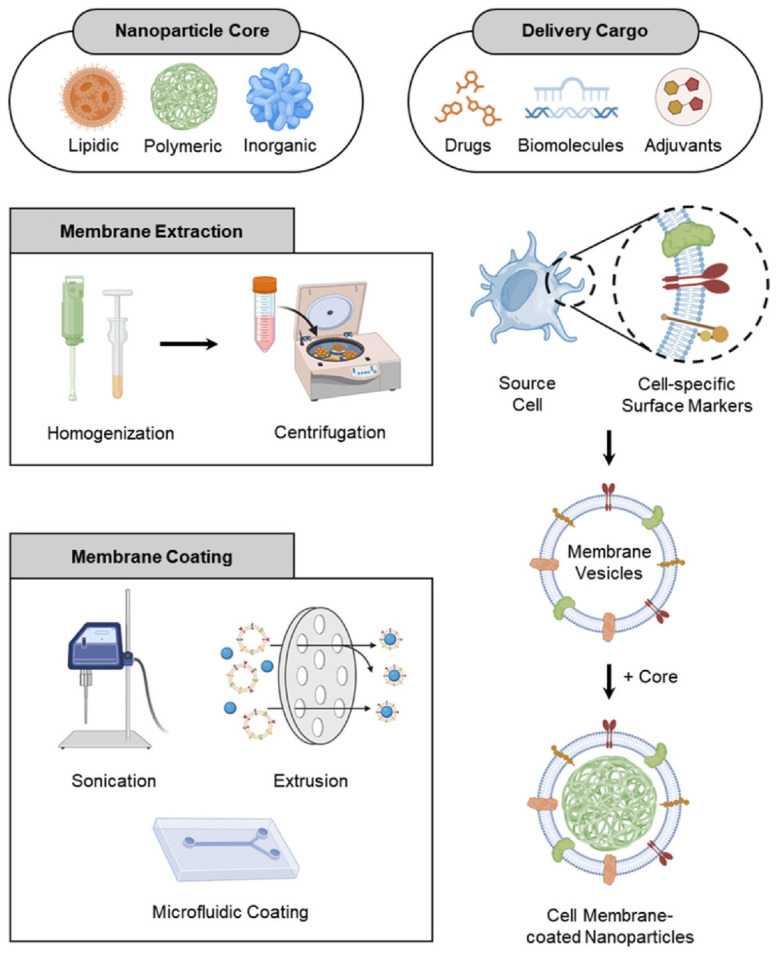
A schematic outline of the steps involved in creating CMNPs [52]. These steps include isolating the membrane, selecting the nanoparticle core (together with its loaded cargo), and subsequently coating the nanoparticle core with the membrane.

The fabrication of cell membrane-coated nanoparticles (CMNPs) generally involves two steps: isolation of membranes from a defined biological source and coating preformed nanoparticle cores with those membranes (Figure 1) [52]. Membrane isolation commonly relies on physical disruption methods such as hypotonic lysis, extrusion, sonication, or freeze–thaw cycling [53,54]. Extrusion produces nanoscale vesicles with relatively controlled size distribution, but may also lead to partial loss of membrane lipids or proteins [55]. Sonication uses acoustic energy to disrupt cellular membranes efficiently, although excessive energy input may damage membrane structure and associated proteins [56,57]. Freeze–thaw cycling is generally milder, but repeated cycling may reduce preparation efficiency [58]. Commercial isolation kits are also used, especially for extracellular vesicle purification, but their cost limits large-scale application [59]. In practice, membrane isolation methods must balance membrane integrity, yield, and downstream application requirements.

Multiple coating strategies have been developed to assemble membrane vesicles with synthetic nanoparticle cores, and each presents distinct advantages and limitations. Physical extrusion remains one of the earliest and most widely used coating methods [23,60]. In this method, nanoparticle cores and membrane vesicles are co-extruded through porous membranes, allowing membrane fragments to reorganize around the core and form core–shell structures. Extrusion can preserve membrane functionality and achieve good coating efficiency, but the multistep process increases material loss and remains difficult to scale. Sonication-based fusion is a commonly used alternative in which ultrasonic energy promotes membrane–nanoparticle fusion and generates relatively uniform nanostructures [54,61]. It is rapid and can reduce membrane waste, but it requires careful control to avoid damaging membrane proteins. Electrostatic interaction-driven coating exploits attractive forces between charged nanoparticles and membrane vesicles [62]. This approach can support membrane attachment, although optimization is still needed to avoid premature dissociation under physiological conditions [63]. In addition to these conventional approaches, several newer fabrication strategies have been developed. Microfluidic electroporation combines microscale fluid handling with short high-voltage pulses to transiently permeabilize membrane vesicles and enhance fusion with nanoparticle cores [52]. Such systems can improve membrane coverage, particle uniformity, and batch reproducibility while reducing membrane protein damage [64,65]. However, optimization of pulse voltage, duration, and flow conditions remains essential, and the technical complexity and cost still limit broader application [66]. Biologically driven in situ packaging strategies have also recently been developed [67]. In this approach, living cells internalize nanomaterials such as iron oxide, gold nanoparticles, or quantum dots and later secrete membrane vesicles containing the incorporated materials under controlled culture conditions. This strategy avoids separate membrane purification and offers an alternative route for generating membrane-derived nanovesicles.

### 2.3. Current Technical Limitations and Emerging Solutions

Although CMNP engineering has advanced rapidly, several technical limitations still restrict reproducibility, scalability, and mechanistic control [16,68]. A central challenge is preserving membrane integrity and protein orientation during coating, because loss or inversion of key surface proteins may compromise biological activity. Orientation-controlled assembly and affinity-guided anchoring strategies have been explored to improve outward presentation of functional membrane proteins and maintain targeting or immune-evasive functions [69].

Another limitation is that natural membranes are not always sufficiently standardized or tunable for specific therapeutic purposes. Genetic engineering of donor cells offers one way to address this problem by introducing defined receptors or immunomodulatory molecules prior to membrane isolation [70]. This strategy can convert CMNPs from passive biomimetic carriers into more controllable systems with tunable biological functions. Manufacturing scalability and batch reproducibility remain major barriers to clinical translation. Microfluidic-assisted and continuous manufacturing technologies have been developed to improve process control and production consistency [71]. Overall, current work in this area is moving toward more standardizable and engineerable CMNP platforms, but further progress will require better control over membrane orientation, coating completeness, batch consistency, and functional quality assessment.

## 3. Major Membrane Sources and Their Functional Roles in CMCT

**Figure 2:**
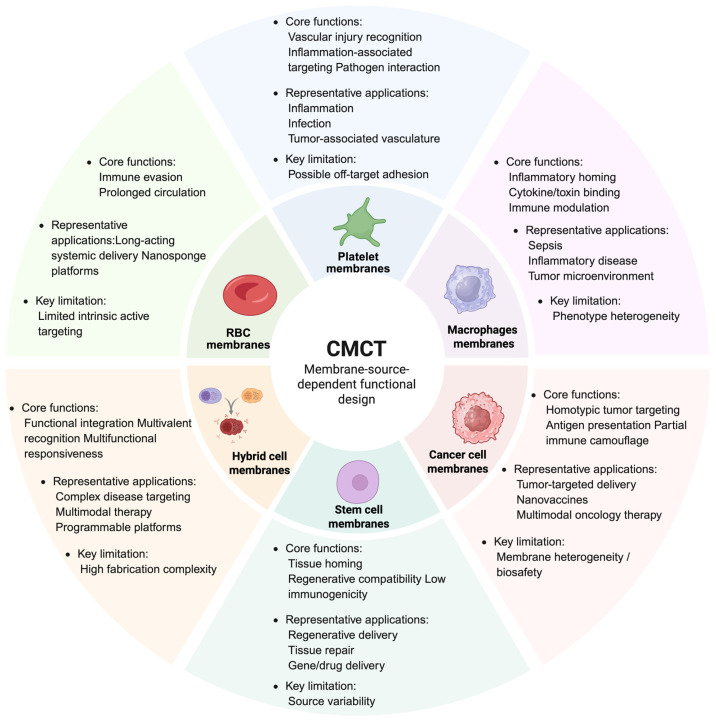
Functional logic of membrane source selection in CMCT. Different membrane sources confer distinct biological functions on cell membrane-coated nanoparticles, including prolonged circulation, vascular injury recognition, inflammatory homing, homologous tumor targeting, regenerative compatibility, and multifunctional integration. This schematic summarizes the core functions, representative applications, and key limitations associated with major membrane sources used in CMCT, highlighting the principle that membrane selection should be matched to the therapeutic task.

The main advantage of CMCT lies in its ability to transfer selected biological functions from natural cell membranes onto synthetic nanocarriers [72,73]. Different membrane sources carry distinct protein, receptor, and glycosylation profiles, which largely determine the in vivo behavior of membrane-coated nanoparticles (Table 1). Figure 2 further illustrates the functional logic of membrane source selection in CMCT, highlighting how different membrane types are matched to distinct therapeutic tasks such as prolonged circulation, inflammatory targeting, tumor homing, regenerative delivery, and multifunctional integration. A major challenge in nanomedicine remains the rapid recognition and clearance of exogenous nanoparticles by the MPS. After entering the circulation, nanoparticles often adsorb plasma proteins and form a protein corona, which alters surface identity and promotes uptake by phagocytic cells in the liver and spleen [74,75]. For this reason, immune evasion, lesion recognition, and microenvironment responsiveness have become key design goals in CMCT-based systems [76,77].

**Table 1:** Major membrane sources used in CMCT and their representative functional features.

Membrane Source	Main Functional Features	Typical Strengths in CMCT	Typical Limitations	References
Red blood cell (RBC)	CD47-mediated self-recognition; low complement activation; prolonged circulation	Immune evasion; extended systemic exposure; nanosponge applications	Limited intrinsic active targeting; often requires secondary engineering	[23,74,78,79,80,81,82]
Platelet	Adhesion to activated vasculature; inflammatory accumulation; pathogen interaction	Vascular injury targeting; inflammation targeting; microbial/toxin binding	Possible off-target adhesion; source variability; scale-up challenges	[46,83,84,85,86]
Macrophage	Inflammatory homing; cytokine/toxin interaction; phenotype-dependent functionality	Inflammation targeting; immune modulation; nanosponge strategies	Phenotype heterogeneity; source standardization; scale-up complexity	[87,88,89,90,91,92,93]
Cancer cell	Homotypic recognition; tumor-antigen carriage; partial immune camouflage	Tumor targeting; nanovaccine potential; multimodal oncology integration	Membrane heterogeneity; biosafety concerns; preparation consistency	[94,95,96,97,98,99]
Stem cell	Tissue homing; low immunogenicity; regenerative compatibility	Lesion localization; regenerative applications; flexible engineering potential	Membrane variability; source consistency; biosafety and manufacturing concerns	[100,101,102,103,104,105,106,107,108,109]
Hybrid membrane	Combination of complementary functions from multiple cell types	Multifunctionality; integration of circulation, targeting, and immune modulation	High fabrication complexity; quality-control burden; reproducibility challenges	[31,110,111,112,113,114]

### 3.1. Red Blood Cell (RBC)

**Figure 3:**
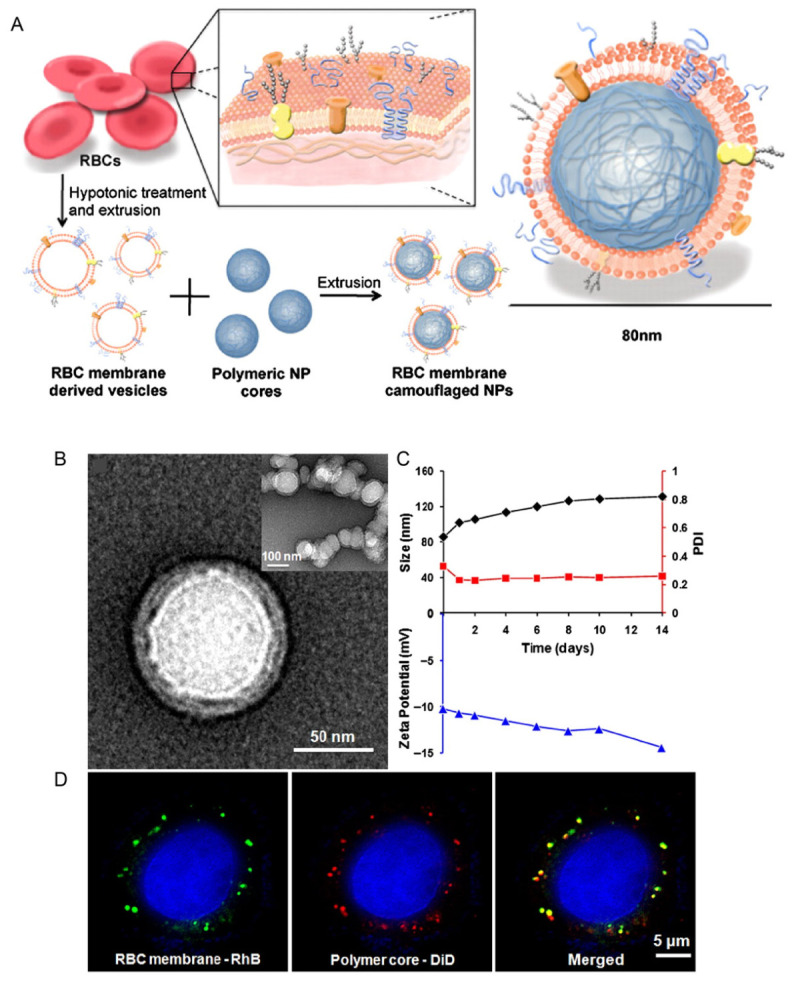
Red blood cell (RBC)-membrane-coated PLGA NPs for prolonged systemic circulation and immune evasion [23]. (**A**) Schematics of the preparation process of the RBC-membrane-coated PLGA NPs. (**B**) The NPs were negatively stained with uranyl acetate and subsequently visualized with TEM. (**C**) DLS measurements of the size, PDI, and surface zeta potential of the nanoparticles over 14 d. (**D**) Scanning fluorescence microscopy images demonstrated the colocalization of the RBC membranes (visualized with green rhodamine-DMPE dyes) and polymeric cores (visualized with red DiD dyes) after being internalized by HeLa cells for 6 h. The excess nanoparticles were washed out, and the cells were subsequently fixed for imaging, indicating that the nanoparticles retained an intact core–shell structure after cellular uptake.

RBC membranes are among the earliest and most widely studied membrane sources in CMCT, primarily because they provide a simple and effective strategy for prolonging circulation. Mature RBC circulate in the bloodstream for approximately 120 days and express multiple immunoregulatory proteins on their surfaces such as CD47, CD55, and CD59, which help limit immune clearance [74]. In particular, CD47 provides a well-known “don’t eat me” signal through interaction with SIRPα on macrophages [44,115]. This makes RBC membranes especially useful when the main design goal is immune evasion and extended systemic exposure [77]. A landmark study in 2011 established RBC membranes as a proof-of-concept coating material for biomimetic nanoparticles [23]. In that study, intact RBC membranes were transferred onto synthetic nanoparticle cores, preserving the major membrane lipids, proteins, and glycoproteins. The use of RBC membranes as an initial proof-of-concept model established the basis for subsequent expansion of membrane-engineered nanosystems derived from diverse cell types (Figure 3) [23]. After coating, retained membrane proteins, glycans, and lipid bilayer components help preserve erythrocyte-derived immune-evasive properties [78]. RBC membrane–coated nanoparticles show reduced macrophage uptake and markedly prolonged circulation compared with PEGylated counterparts [23]. As a result, they can improve passive accumulation in diseased tissues even without additional ligands.

Beyond CD47-mediated signaling, RBC membranes also help reduce complement activation and nonspecific protein adsorption, which further limits acute immune recognition after systemic administration [23,63]. Compared with unmodified synthetic nanomaterials, RBC-coated systems generally show lower complement deposition and reduced macrophage sequestration [79]. Compared with PEGylation, membrane camouflage may also avoid anti-PEG antibody generation and the associated accelerated blood clearance phenomenon, which is relevant for repeated or long-term administration [116].

In addition to improving pharmacokinetics, RBC membranes can support other therapeutic functions through preserved membrane-associated proteins. A representative extension of this strategy is the use of RBC-coated nanoparticles as biomimetic “nanosponges” for binding bacterial toxins or inflammatory mediators in infectious and inflammatory diseases [81]. More recent studies have further expanded RBC membrane-based carriers into multifunctional systems combined with targeting ligands, phototherapy, or immune-modulating components [82,117]. Overall, RBC membranes remain best suited to applications in which immune camouflage and extended circulation are the main properties, although additional engineering is usually required when strong active targeting is needed.

### 3.2. Platelets

Platelet membranes are of particular interest in CMCT because platelets naturally participate in hemostasis, vascular repair, inflammation, and tumor-associated interactions [118]. Their biological value comes from membrane proteins that mediate adhesion to activated endothelium and injured vasculature, including integrins, glycoprotein complexes, and selectin-related pathways. Through interactions with ligands such as von Willebrand factor (vWF), intercellular adhesion molecule-1 (ICAM-1), and vascular cell adhesion molecule-1 (VCAM-1), platelets can adhere, roll, and accumulate at sites of endothelial activation [119,120]. This behavior is especially relevant in inflammatory and tumor microenvironments, where vascular activation promotes selective platelet accumulation. As a result, platelet membranes are particularly useful when vascular injury recognition or inflammation-associated targeting is required.

Based on these properties, platelet membrane–coated nanoparticles (PM-NPs) can transfer platelet-derived recognition and adhesion functions to synthetic nanocores. A landmark study showed that coating nanoparticle cores with platelet membranes could preserve key surface proteins and enable recognition of injured vasculature and microbial targets in vivo [46]. Compared with single-ligand modification strategies, platelet membrane coating provides a broader and more adaptable recognition interface without requiring additional chemical conjugation. Because platelets are autologous circulating components, their membranes also retain self-recognition molecules such as CD47, CD55, and CD59, which help reduce MPS-mediated clearance [44,121]. Accordingly, platelet membrane coating can also improve circulation behavior and reduce hepatic and splenic uptake [46,83]. Beyond vascular targeting, platelet membranes can also function as pathogen-interacting interfaces through preserved membrane receptors [122]. Representative studies have shown that PM-NPs can bind pathogens such as *Staphylococcus aureus* and help neutralize secreted toxins in infection models [46]. Similar systems have also been used as biomimetic nanosponges to sequester toxins and attenuate inflammation in sepsis-related settings [80].

Platelet membranes are also relevant in cancer because platelet–tumor interactions contribute to vascular adhesion, immune evasion, and metastatic dissemination [123,124]. This has led to platelet membrane–based nanocarriers designed to target both circulating tumor cells and tumor-associated vasculature. Representative examples include platelet membrane–coated doxorubicin nanoparticles that enhance drug accumulation at tumor-associated sites and suppress metastasis [83]. Platelet membranes have also been combined with photothermal and photodynamic platforms for multimodal cancer therapy [84]. In addition, platelet membrane–based systems have been explored for targeted delivery and immune modulation in inflammatory diseases. In intestinal inflammation, representative studies have shown that platelet membrane–coated nanoparticles can alleviate ulcerative colitis by interfering with platelet–immune interactions and promoting mucosal repair [85,86]. Hybrid membrane strategies have further expanded the functional range of platelet-based systems. For example, fusion with cancer cell membranes can combine vascular recognition with homologous tumor targeting, whereas integration with RBC membranes can add prolonged circulation to platelet-derived adhesion functions [29,31]. These combinations illustrate how platelet membranes can be incorporated into more modular and programmable CMCT designs. Overall, platelet membranes are best suited to settings in which vascular recognition, inflammatory targeting, or pathogen interaction is needed, while their broader clinical translation will still depend on better control of source consistency, biosafety, and manufacturing scalability.

### 3.3. Macrophage

Macrophage membranes are widely studied in CMCT because macrophages naturally participate in inflammation, immune surveillance, and tissue remodeling. Their value in biomimetic delivery comes from adhesion molecules and chemokine receptors that support migration toward inflamed or diseased tissues [125,126]. These interactions enable adhesion and transendothelial migration, providing the basis for inflammatory homing. By leveraging these surface features, macrophage membrane–coated nanoparticles (MM-NPs) can preferentially localize to inflammatory and, in some cases, tumor microenvironments [127]. Compared with conventional ligand-conjugated systems, this biomimetic strategy offers a broader, multivalent recognition interface without additional chemical modification. In addition, self-recognition markers such as CD47 may help reduce MPS-mediated clearance and improve circulation behavior [127].

Beyond inflammatory homing, macrophage membranes also provide immune-interactive interfaces through preserved pattern-recognition and cytokine-binding functions [89]. Accordingly, MM-NPs have been explored in disease settings characterized by endothelial activation and high inflammatory burden, including tumors, sepsis, and cardiovascular disorders [87,88,89]. Because macrophage membranes can retain pathogen-binding and cytokine-interacting components, they are also well suited to “nanosponges” strategies aimed at adsorbing endotoxins or inflammatory mediators [87]. An additional feature of macrophage membranes is phenotype-dependent functionality: M1-derived membranes may favor pro-inflammatory or antitumor effects, whereas M2-derived membranes are more closely associated with immune regulation and tissue repair [126]. This has enabled some degree of functional tuning through polarization-specific membrane selection in both inflammatory and anticancer applications [89,90].

**Figure 4:**
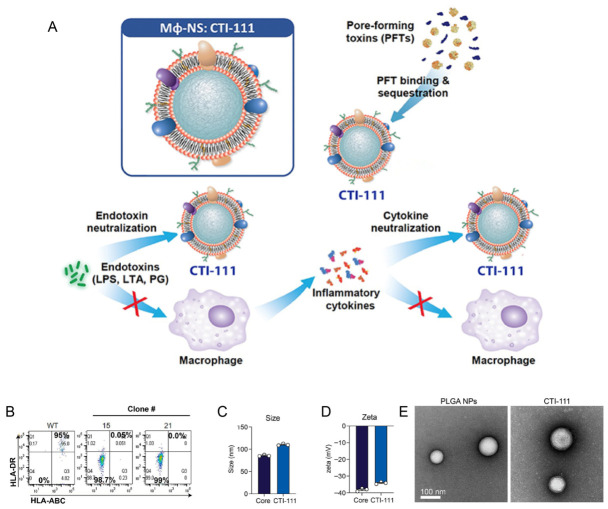
Macrophage membrane–coated nanoparticle (MM-NPs) for toxin and cytokine neutralization in sepsis [93]. (**A**) MM-NPs, exemplified by CTI-111, function as biomimetic nanodecoys capable of simultaneously sequestering microbial toxins and pro-inflammatory cytokines, thereby attenuating systemic inflammation and improving survival in experimental sepsis models. (**B**) HLA expression of candidate clones performed by Bio-Techne, Inc. Expression of HLA-ABC and HLA-DR was detected by flow cytometry. (**C**) Uncoated PLGA cores (black) and CTI-111 (blue) hydrodynamic diameter (nm) and (**D**) zeta potential (mV) were measured via dynamic light scatter (n = 3). (**E**) Transmission electron microscopy (TEM) images of PLGA nanoparticle cores and CTI-111 after coating with macrophage membranes. Scale bar 100 nm.

Recent studies have positioned MM-NPs as multifunctional biomimetic platforms for inflammatory and immune-related disease intervention. In infectious and inflammatory diseases, macrophage membrane coatings have been used to improve site-specific delivery and modulate pathogenic immune responses [91,92]. A representative example is CTI-111, a macrophage membrane-coated nanoparticle developed as a decoy platform. This system was designed to neutralize both bacterial toxins and pro-inflammatory cytokines in a pathogen-independent manner. In murine sepsis models, it reduced systemic inflammation and improved survival, illustrating the therapeutic potential of macrophage-derived nanosponges [93] (Figure 4). Similar approaches have also been explored in oncology and in hybrid membrane systems, where macrophage membrane-derived functions are combined with other targeting or immunomodulatory features [18]. Overall, macrophage membranes are particularly valuable when inflammatory homing and broad immune interaction are required within the same platform. Their main strengths are functional breadth and disease-site responsiveness, although phenotype heterogeneity and membrane standardization remain important challenges for further translation [20,69,127].

### 3.4. Cancer Cell

Cancer cell membranes are mainly used in CMCT when homotypic tumor targeting is a primary objective, owing to their intrinsic ability to recognize tumors of the same cellular origin and their relative accessibility in vitro. Their value comes from preserved adhesion molecules and tumor-associated antigens, including cadherin, integrins, epithelial cell adhesion molecule (EpCAM), CD44, and other membrane-associated glycoconjugates. These surface components support homotypic recognition among tumor cells and can promote selective retention within tumor microenvironments [94]. Tumor cell membranes may also retain immune-regulatory molecules such as CD47, which can help reduce phagocytic clearance. As a result, cancer cell membranes provide dual advantages of homotypic targeting and partial immune camouflage, making them attractive templates for tumor-oriented biomimetic delivery systems [128,129].

Based on these properties, cancer cell membrane–coated nanoparticles (CCM-NPs) have been developed as tumor-oriented biomimetic delivery platforms. In 2014, Liangfang Zhang and colleagues demonstrated that tumor cell membrane–coated PLGA nanoparticles could support both vaccine-like antigen delivery and homologous tumor targeting [94]. These systems retained tumor-derived adhesion molecules and antigens, leading to improved tumor homing in vivo. Compared with single-ligand strategies, cancer cell membranes provide a broader recognition interface composed of multiple native membrane components [20]. This may improve adaptability in heterogeneous tumor microenvironments, where single-target approaches often perform inconsistently. In addition, retention of membrane-associated regulatory proteins may help reduce macrophage uptake and support circulation stability [130]. This allows homologous targeting to be combined with passive tumor accumulation, improving local drug enrichment. Beyond targeted delivery, cancer cell membranes can also serve as endogenous carriers of tumor antigens, enabling nanovaccine design. Because tumor-derived membranes contain broad antigen repertoires, they may support dendritic cell processing and multi-epitope T-cell responses. Representative studies have shown that leukemia cell membrane–derived nanovaccines can induce antigen-specific immune activation in animal models [95]. Related studies have further explored strategies to enhance neoantigen presentation and CD8^+^ T-cell responses in personalized vaccine settings [96]. Collectively, these findings suggest that cancer cell membranes can function not only as targeting shells but also as immunologically active biointerfaces for antigen delivery.

**Figure 5:**
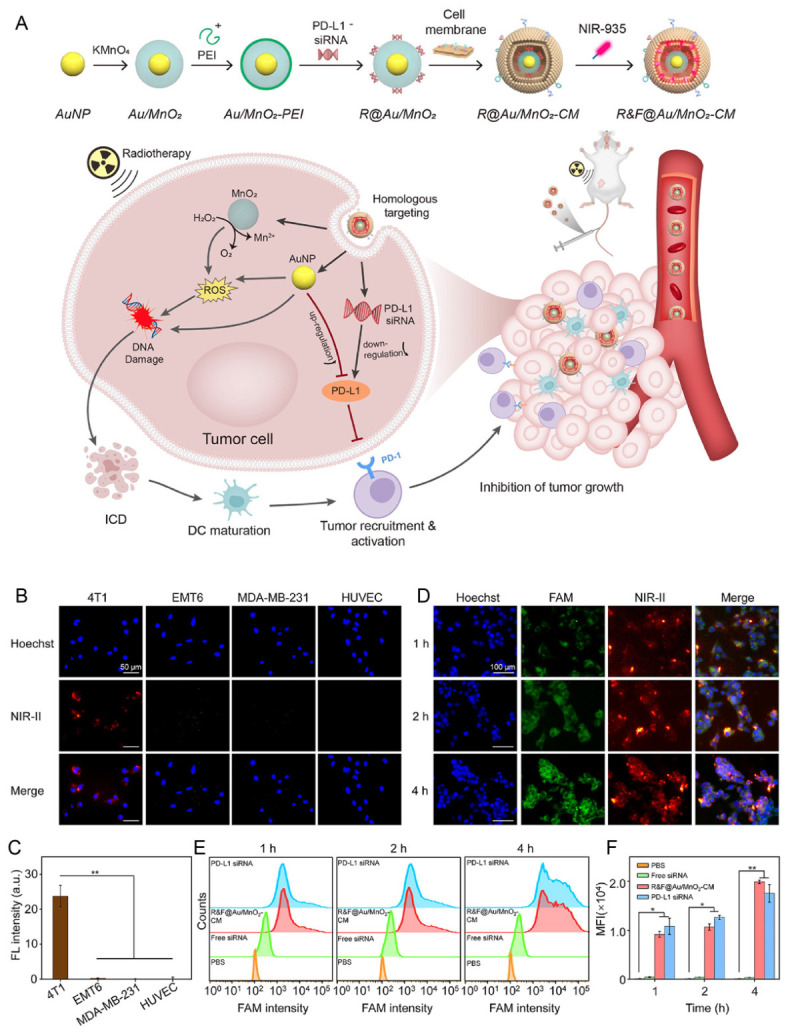
Cancer cell membrane-coated Au/MnO_2_ nanosensitizers for enhanced radio-immunotherapy in breast cancer. (**A**) Schematic illustration of R&F@Au/MnO_2_-CM for cancer-cell-biomimetic nano-radiotherapy sensitizer for synergistically enhanced radio-immunotherapy of breast cancer [99]. In vitro cellular internalization. (**B**) Fluorescence images of cellular uptake of R&F@Au/MnO_2_-CM in different cell lines for 1 h. (**C**) Quantification analysis for the homologous targeting efficiency. (**D**) Fluorescence imaging of 4T1 cells after incubating with R&F@Au/MnO_2_-CM for different time. (**E**) Flow cytometry analysis for the transfection efficiency on 4T1 cells at 1 h, 2 h and 4 h after different treatments. (**F**) Quantification analysis of FCM analysis. Data are given as mean ± SD (n = 3). * *p* < 0.05, ** *p* < 0.01.

Cancer cell membrane-based systems have since been extended into multimodal therapeutic platforms, including chemotherapy, phototherapy, and combined immunotherapy. Representative studies have combined cancer cell membrane-coated nanoplatforms with imaging-guided or energy-assisted therapeutic strategies to improve tumor inhibition [97]. Structural innovations such as yolk–shell architectures improve tumor penetration and therapeutic efficiency [98], while homologous targeting combined with programmed death-ligand 1 (PD-L1) small interfering RNA delivery enhances immunotherapeutic sensitization [99] (Figure 5). Hybrid membrane strategies have further broadened the functional scope of these systems. For example, fusion with dendritic cell membranes can combine tumor targeting with antigen presentation, whereas fusion with RBC membranes can improve circulation while preserving homologous recognition [131,132]. These examples show how cancer cell membranes can be integrated into more flexible and multifunctional CMCT designs. Overall, cancer cell membranes are particularly attractive when both tumor-oriented targeting and antigen-related immune engagement are desired. Their main strengths are broad tumor recognition and immunological relevance. They are also unusual in that they can support both drug-delivery and vaccine-oriented strategies within the same platform. However, membrane heterogeneity, biosafety, and preparation consistency remain major translational concerns.

### 3.5. Stem Cell

Stem cell membranes are of interest in CMCT because stem cells naturally respond to chemotactic cues and migrate toward pathological sites, especially within tumor and inflammatory microenvironments [100]. Among the available sources, mesenchymal stem cells (MSC) are the most widely used because of their low immunogenicity and relative ease of expansion [100]. However, direct use of living stem cells for delivery remains limited by safety concerns, including uncontrolled differentiation and unpredictable biological behavior. In contrast, stem cell membranes can retain useful biological functions while avoiding many of the risks associated with administering live cells. Their value comes from preserved chemokine receptors and adhesion molecules, such as C-X-C chemokine receptor type 4 (CXCR4), CCR2, CD44, and integrin-related pathways, which support transendothelial migration and site-specific recruitment [133,134,135]. As a result, coating nanocarriers with stem cell membranes can preserve tissue-homing capability and immune compatibility while improving nanocarrier localization and circulation behavior. Induced pluripotent stem cell technologies may further expand the availability of more standardized membrane sources for biomimetic platform construction [101].

The relatively low immunogenicity also supports stable performance across heterogeneous pathological environments [100]. Stem cell membrane–engineered nanoplatforms have been explored in tumor-targeted therapy, controlled drug delivery, and combination treatment strategies, often with improved lesion accumulation. Reconstitution studies have combined stem cell membranes with nanogels or functional nanoparticles to improve systemic stability and support stimulus-responsive drug release [102,103,104]. Beyond oncology, stem cell membrane-coated nanoparticles (SCM-NPs) have also attracted growing interest in tissue engineering and regenerative medicine. Representative examples include systems designed to improve targeting to bone defect sites and support osteogenic repair [105]. Similar strategies have also been explored for osteoarthritis and other regenerative settings that require lesion localization and tissue repair support [106]. Stem cell membrane platforms have additionally been investigated for chemotherapeutic and clustered regularly interspaced short palindromic repeats (CRISPR)-Cas9-based delivery, highlighting their potential versatility across disease models [107,135].

Recent work has increasingly focused on engineering stem cell membranes to create more programmable biomimetic interfaces. Strategies such as fusion with targeting lipids, or incorporation of engineered components have been used to retain homing properties while improving targeting precision and therapeutic control [106,108,109]. Hybrid membrane designs have further expanded this concept by combining stem cell membranes with RBC, immune-cell, or tumor-cell membranes to integrate homing with other complementary functions [136,137,138]. These approaches move stem cell membrane platforms beyond passive targeting toward more multifunctional engineered delivery interfaces. Overall, stem cell membranes are particularly attractive when tissue homing and regenerative compatibility are desired without the risks of live-cell administration. Their main strengths are biological adaptability, lesion localization, and compatibility with regenerative applications. However, membrane compositional variability, manufacturing consistency, and long-term biosafety remain important barriers to broader translation.

### 3.6. Hybrid Cell Membrane

Hybrid membrane systems were introduced in CMCT because a single membrane source is often insufficient to provide all desired biological functions at once. Early work with single-source membranes, such as RBC-coated nanoparticles, demonstrated that membrane transfer could endow particles with useful biological functions [23]. This, in turn, raised the possibility of combining functions from multiple membrane sources within a single nanosystem. As different membrane sources were found to offer distinct strengths, such as prolonged circulation, inflammatory homing, homologous targeting, or immune modulation, hybrid membrane strategies emerged as a logical extension.

In practice, many disease settings require both circulation stability and precise biological recognition, which single-source membranes do not always provide at the same time. Hybrid membrane fusion was therefore proposed as a way to integrate complementary functions from multiple cell types into one platform. A representative example is the RBC–platelet hybrid system, which combines RBC-derived immune camouflage with platelet-mediated pathological targeting [31]. Subsequent studies extended this idea to a broader range of dual- and multi-source membrane systems. For example, RBC–tumor hybrids can combine prolonged circulation with homologous tumor recognition, whereas incorporation of macrophage- or leukocyte-derived components can add inflammatory homing and transendothelial migration features [139,140]. Compared with single-membrane strategies, hybrid membranes can provide a broader functional profile. Their main attraction lies in the ability to coordinate multivalent recognition with improved disease-site responsiveness.

More recently, hybrid membrane design has moved beyond simple functional combination toward more programmable biointerface engineering. Designs have expanded from binary fusion to more complex architectures capable of integrating circulation support, active targeting, and immune modulation within the same nanosystem [141]. Genetic engineering and membrane-surface modification strategies have further been used to regulate key membrane functions and improve recognition specificity or therapeutic responsiveness [110]. These advances have widened the use of hybrid membranes in multimodal therapeutic systems, including chemo-immunotherapy, phototherapy, and nucleic acid delivery [142]. Overall, hybrid membrane systems represent the most functionally flexible branch of CMCT. Their main advantage is the ability to integrate complementary programs from different cell types within one delivery interface [112,113,114]. When combined with molecular engineering, they can move beyond passive biomimicry toward more responsive and multifunctional delivery platforms [111]. However, this added flexibility comes at the cost of greater fabrication complexity, more demanding quality control, and increased challenges in reproducibility and standardization.

## 4. Application of CMNPs in the Treatment of Intestinal Diseases

Effective intestinal nanodelivery requires overcoming multiple biological barriers, including gastrointestinal acidity, enzymatic degradation, mucus obstruction, epithelial tight junctions, and dynamic modulation by gut microbiota and inflammatory factors. In addition, systemically administered nanoparticles are frequently subjected to rapid clearance by the MPS, limiting accumulation at diseased intestinal sites [143]. CMNPs help address these limitations by reconstructing biologically derived interfaces that support immune evasion, inflammation recognition, and lesion-specific accumulation. They may also partially protect nanocarriers from gastrointestinal destabilization. These properties provide advantages for treating inflammatory bowel disease (IBD), colorectal cancer, and intestinal infections.

However, intestinal microenvironment heterogeneity and challenges in membrane sourcing, protein orientation control, and scalable manufacturing continue to hinder clinical translation. Recent engineering strategies therefore integrate membrane biomimicry with stimulus-responsive materials and hybrid membrane design to improve delivery robustness in complex intestinal environments.

### 4.1. Inflammatory Bowel Disease (IBD)

IBD is characterized by persistent immune dysregulation and disruption of intestinal barrier homeostasis. These changes are accompanied by continuous recruitment of inflammatory cells and sustained activation of the local microenvironment [144]. Effective therapeutic delivery therefore requires not only selective accumulation at inflamed intestinal sites but also coordinated regulation of immune responses and mucosal repair. CMNPs offer a biomimetic strategy for inflammation-directed delivery by inheriting the chemotactic and recognition properties of immune cells. Collectively, CMNP-based strategies have evolved from inflammation homing and inflammatory mediator sequestration toward oxidative stress regulation and gene-level intervention. This progression allows therapeutic modulation across the initiation, amplification, and resolution phases of intestinal inflammation.

CMNP-based therapies for IBD have progressively transitioned from passive drug carriers toward bioinspired platforms capable of multistage inflammatory regulation. Membranes derived from immune cells retain intrinsic inflammatory homing properties, enabling active localization within diseased intestinal tissues. Macrophage membrane–coated nanosystems exploit surface chemokine receptors and adhesion molecules to promote accumulation in inflamed colon regions. They can also adsorb pro-inflammatory cytokines and promote macrophage polarization from the M1 to M2 phenotype, thereby suppressing immune overactivation and facilitating mucosal repair [145]. Integration of reactive oxygen species (ROS)-responsive materials or CRISPR/Cas9-based systems further enables inflammation-triggered drug release and targeted regulation of pathogenic molecules such as CD98 [146]. This extends therapeutic intervention toward molecular-level modulation. Neutrophil membrane–engineered nanoparticles exploit rapid inflammatory recruitment and transendothelial migration mechanisms to enhance lesion localization [147]. When combined with anti-inflammatory agents or antioxidant nanozymes, they can also mitigate oxidative stress–induced epithelial injury. Similarly, platelet membrane–inspired nanosystems interfere with platelet–immune cell interactions through blockade of the P-selectin/PSGL-1 axis [148]. This can attenuate inflammatory signal amplification and support intestinal barrier restoration.

**Figure 6:**
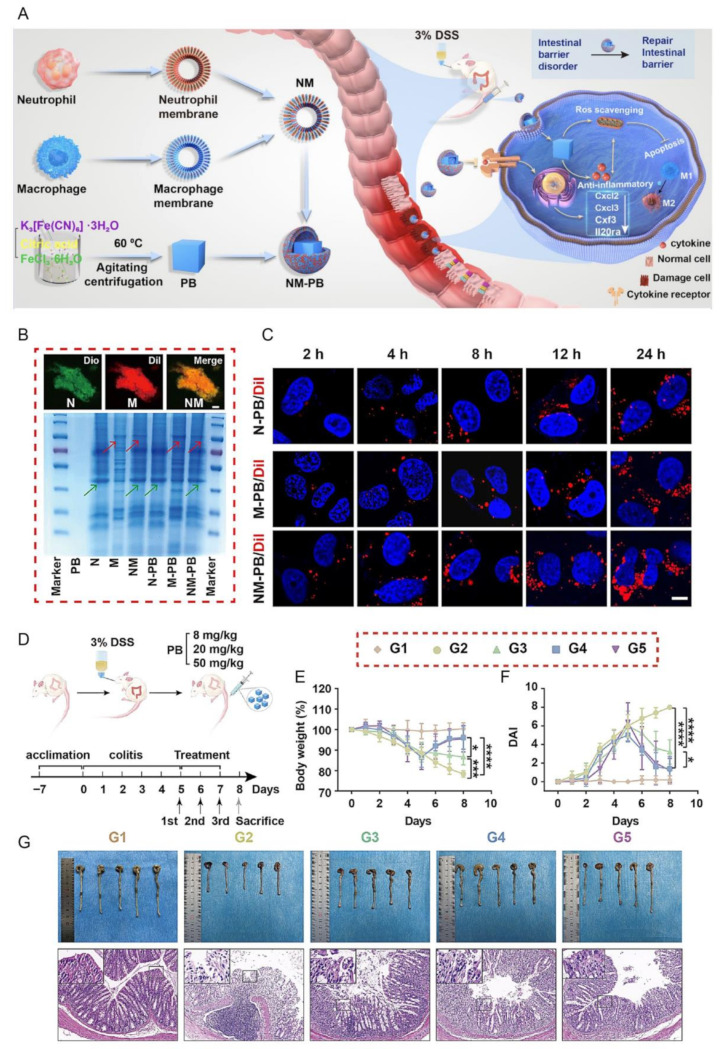
Neutrophil–macrophage hybrid membrane-coated Prussian blue (NM-PB) nanozymes for ulcerative colitis therapy [152]. (**A**) A schematic representation illustrating the preparation process of NM-PB nanozymes and their functional impacts in UC mouse models. (**B**) CLSM images of the neutrophil-macrophage hybrid membrane (scale bar = 10 μm); SDS-PAGE protein analysis of PB nanozymes, N, M, NM, N-PB nanozymes, M-PB nanozymes, and NM-PB nanozymes by Coomassie blue staining. Coomassie brilliant blue staining analysis revealed that the protein bands observed on NM-PB were comparable to those of isolated N and M fractions, as indicated by the green and red arrows, thereby indicating the presence of membrane proteins. (**C**) Intracellular uptake of N-PB, M-PB, and NM-PB nanozymes (the blue indicates nucleus stained with DAPI, the red indicates N-PB, M-PB, and NM-PB nanozymes stained with Dil) (scale bar =  20 μm). Determination of the optimal dosage of PB nanozymes for the treatment of UC model in mice. (**D**) Schematic representation of the experimental protocol employed for UC treatment using PB nanozymes. (**E**) Changes in body weight following various treatments. (**F**) Disease Activity Index (DAI) changes after different treatments. (**G**) Representative images illustrating the macroscopic appearance and H&E staining of colon tissue after different treatments (scale bar = 200 μm). * *p*  < 0.05, *** *p*  < 0.001, **** *p*  < 0.0001. one-way ANOVA, Tukey’s multiple comparison test.

Beyond single-membrane approaches, engineered and hybrid membrane strategies further expand the therapeutic scope of biomimetic nanomedicine for IBD. Leukocyte-derived leukosome systems enable competitive inhibition of immune cell recruitment through integrin-mediated targeting of inflamed vasculature. By contrast, membrane nanosponges can neutralize endotoxins and inflammatory mediators through intrinsic membrane-binding interactions [149,150]. Recent studies increasingly incorporate genetic engineering and membrane functional reprogramming to enhance targeting precision. For example, macrophage membranes engineered to overexpress Toll-like receptor 4 (TLR4) improve inflammatory colon recognition and regulate the S100A9 signaling axis to alleviate colitis progression [146]. CRISPR/Cas9-loaded biomimetic metal–organic framework (MOF) platforms further enable site-specific editing of pathogenic targets such as CD98 [151]. This extends intervention from inflammatory suppression toward gene-level therapy. Hybrid immune cell membrane systems integrating neutrophil and macrophage functionalities simultaneously enhance chemotaxis, immune evasion, and ROS scavenging, thereby promoting immune reprogramming and intestinal barrier repair [152] (Figure 6).

CMNP-based strategies in IBD have undergone a mechanistically driven therapeutic evolution, moving from inflammation-directed homing and inflammatory mediator sequestration toward oxidative stress modulation and gene-level intervention. The integration of complementary biological functions derived from distinct immune cell membranes enables CMNPs to modulate multiple stages of intestinal inflammation, including initiation, amplification, and tissue repair. This systems-oriented design provides a conceptual framework for multifunctional therapeutic platforms in IBD. Such platforms may improve disease control while helping to limit systemic toxicity.

### 4.2. Colorectal Cancer (CRC)

Colorectal cancer (CRC) progression is governed by pronounced tumor heterogeneity and an immunosuppressive microenvironment. In this setting, therapeutic resistance often arises from insufficient tumor penetration and limited target recognition. Effective nanodelivery systems must therefore simultaneously overcome circulatory clearance, inefficient tumor localization, and dynamic microenvironmental barriers. CMNPs address these challenges by reconstructing biologically derived recognition interfaces from tumor or blood cells. This allows the integration of homotypic targeting, immune evasion, and multifunctional therapeutic loading. This biomimetic strategy establishes an effective framework for enhancing tumor enrichment and enabling synergistic therapeutic intervention in CRC [20,153,154].

CMNP-based strategies for CRC have evolved from improving circulation stability and tumor retention toward integrating multimodal therapeutic and immune–gene regulatory functions. Early membrane camouflage approaches, particularly platelet-derived coatings, enhanced intratumoral retention and immune interaction [155]. In some systems, this supported localized activation of antitumor immunity through delivery of immunomodulators such as the TLR7/8 agonist R848. Subsequently, tumor cell membrane–mediated homotypic targeting emerged as a central strategy. Biomimetic nanoparticles derived from CRC cell lines (e.g., CT26 or MC38) showed improved tumor recognition and accumulation, while also supporting photothermal or chemotherapeutic interventions [156,157].

Building upon tumor enrichment capability, membrane-engineered nanosystems increasingly incorporate gene and metabolic regulation modules. Platforms delivering miRNA or STAT3-siRNA enable pathway-specific suppression of tumor proliferation and sensitization to chemotherapy [158,159,160]. By contrast, co-delivery systems combining chemotherapeutics and nucleic acids can disrupt angiogenic and survival signaling networks. Integration of energy-based therapies further enhances therapeutic efficacy. For example, cancer membrane–encapsulated Cu_9_S_8_ nanostructures achieve synergistic photothermal–sonodynamic tumor ablation while improving tumor accumulation efficiency [161]. In parallel, macrophage membrane modification strengthens interactions with the tumor immune microenvironment through inflammation-mimicking adhesion and chemotactic behavior, promoting immune-mediated therapeutic synergy [162].

**Figure 7:**
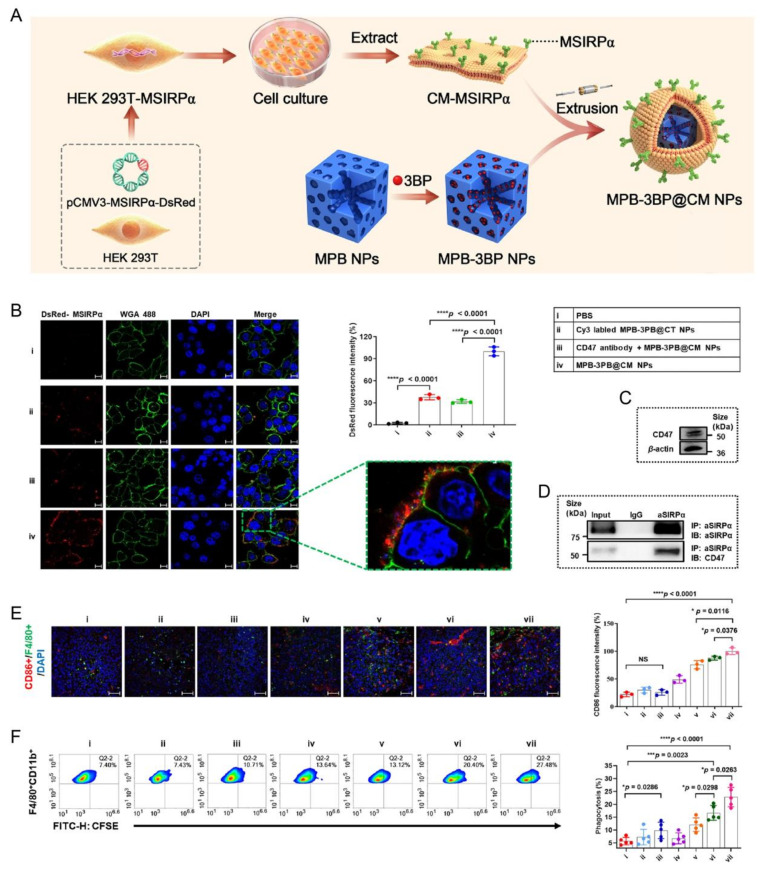
Genetically engineered cell membrane-coated Prussian blue nanoplatforms (MPB-3BP@CM NPs) for colorectal cancer immuno-metabolic photothermal therapy [163]. (**A**) The construction of MPB-3BP@CM NPs and their implementation in combined therapy for CRC. (**B**) CLSM was employed to visualize the binding of MPB-3BP@CM NPs on the cell membrane of HCT116 cancer cells, while quantification of DsRed-MSIRPα fluorescence intensity (%) was performed based on CLSM observations. WGA 488 dye was utilized to detect the HCT116 cell membrane (green channel) and DAPI was used to detect the cell nucleus (blue channel). The red channel fluorescence emission originated from DsRed moieties. The image on the right is an enlarged view of the green boxed region in the image on the left. Scale bar = 10 µm. (**C**) Western blot assay exhibited the expression of human CD47 receptors in the HCT116 cell lysate. (**D**) CO-IP and Western blot were employed to examine the interaction between MSIRPα (on MPB-3BP@CM NPs) and CD47 (on HCT116 cells), immunoblot (IB). (**E**) Representative image and quantitative analysis of immunofluorescence staining of the tumor sections showing infiltrated CD86  +  F4/80+ macrophage cells. Scale bar  =  50 μm. (**F**) Representative flow cytometry plots of CFSE  +  F4/80  +  CD11b+ cells in tumor gating on F4/80  +  CD11b+ cells, the phagocytosis efficiency is represented by the percentage of CFSE  +  F4/80  +  CD11b+ cells in total F4/80  +  CD11b+ cells. All data were presented as mean  ±  SD (n  ≥  3).

Beyond natural membrane mimicry, CRC-directed CMNPs platforms are advancing toward engineered and hybrid biointerfaces with programmable functionality. Genetic engineering of donor cell membranes enables controlled presentation of immune-regulatory proteins [163] (Figure 7). For instance, membranes expressing high-affinity SIRPα variants can disrupt CD47-mediated immune escape and enhance macrophage-mediated tumor clearance. Engineered biomimetic systems have also been integrated with metabolic intervention strategies, including ferroptosis induction via tumor-mimetic Prussian blue nanoplatforms activated by targeted delivery and photostimulation [164]. Furthermore, emerging “Trojan-horse” biomimetic nanomachines exploit membrane tropism to navigate tumor-associated microenvironments [165]. This expands membrane engineering from passive targeting toward more active regulation of tumor ecology. These advances indicate a transition of CMNP-based CRC therapy toward more programmable and hybridized biointerface design.

### 4.3. Intestinal Infections and Microbiome Regulation

Intestinal infectious diseases arise from dynamic interactions between pathogen invasion and host immune responses. These interactions can lead to mucosal injury and disruption of microbial homeostasis. Disease progression is typically driven by coordinated processes including toxin release, inflammatory amplification, and microbiota imbalance. Accordingly, therapeutic strategies have shifted from conventional antimicrobial eradication toward multilevel approaches that integrate toxin neutralization, immune regulation, epithelial protection, and microbiome restoration. CMNPs exploit naturally derived biorecognition interfaces to simultaneously interact with pathogenic virulence factors and host inflammatory responses. This provides a biomimetic framework for multi-target therapeutic intervention within complex intestinal infection environments [166].

**Figure 8:**
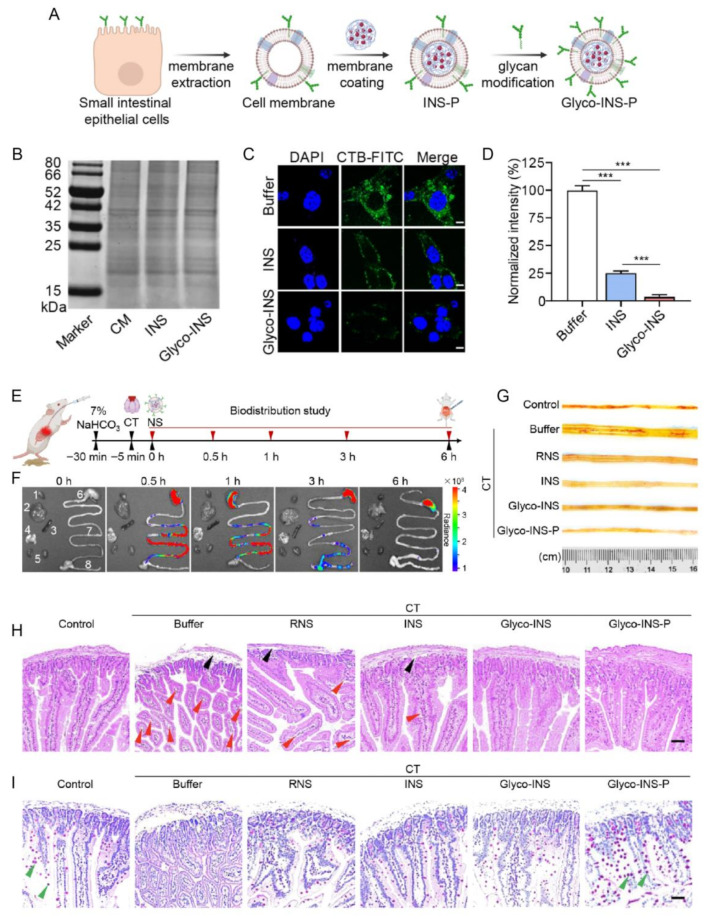
Glycan-modified intestinal epithelial cell membrane-coated nanosponges for cholera toxin neutralization [167]. Schematic illustration of (**A**) glycan-modified biomimetic nanomedicine (Glyco-INS-P) fabrication procedures. (**B**) SDS-PAGE protein analysis of the cell membrane (CM), INS, and Glyco-INS. Fluorescence images (**C**) and normalized intensity (**D**) of intestinal epithelial cells incubated with the remaining CTB-FITC (10 μg/mL) after pretreatment with PBS, INS, and Glyco-INS (1 mg/mL) for 30 min. Scale bars: 5 μm. (**E**) Schematic overview of the experimental timeline for CT detoxification in vivo. (**F**) Biodistribution of DiR-labeled Glyco-INS (10 mg/kg) in the gastrointestinal tract and major organs of CT-treated mice at 0, 0.5, 1, 3, and 6 h after oral gavage. 1, heart; 2, liver; 3, spleen; 4, lung; 5, kidneys; 6, stomach; 7, small intestine; 8, colon. (**G**) Representative segments of the small intestine from CT-treated mice after single-dose oral gavage of various cellular nanosponges (10 mg/kg) for 6 h. Representative H&E (**H**) and PAS (**I**) staining of intestinalsections in different groups. Arrowheads mark edematous submucosa (black), enlarged internal structures (red), and mucin-containing goblet cells (green). Scale bar: 50 μm. Untreated healthy mice were used as control groups. *** *p* < 0.001. Error bars represent the standard deviation (SD, n = 3).

Control of pathogen-derived virulence factors represents a primary intervention level in membrane-based anti-infective therapy. Cell membrane nanosponges function as biomimetic toxin decoys capable of adsorbing bacterial exotoxins through preserved membrane receptors. RBC-derived nanosponges were first shown to neutralize *Staphylococcus aureus* α-hemolysin by mimicking host membrane binding sites, thereby reducing toxin-induced cellular injury [80]. Subsequent glycoengineering of intestinal epithelial membranes further enhanced binding affinity toward cholera toxin while restoring ion transport homeostasis [167] (Figure 8). As a result, therapeutic activity extended beyond toxin sequestration to functional recovery. Beyond detoxification, excessive immune activation induced by endotoxins represents a critical driver of infection-associated pathology. Macrophage membrane–engineered nanoparticles capture lipopolysaccharide and pro-inflammatory cytokines via innate receptors such as TLR4/CD14 [87,168]. This can attenuate inflammatory cascades and limit endotoxemia progression. Advanced multifunctional nanosponges, exemplified by CTI-111, demonstrate broad-spectrum sequestration of toxic and inflammatory mediators [93]. This highlights their translational potential in enteric infection–associated systemic inflammation.

Increasingly, membrane biomimetic strategies extend beyond pathogen elimination toward regulation of host–microbiome interactions. Bacterial OMV-based systems exploit homotypic bacterial recognition to enhance antibiotic delivery against Gram-negative infections [169]. They may also support clearance of both intracellular and extracellular pathogens. In parallel, membrane-inspired platforms have been developed to promote probiotic colonization and microbiota restoration. Macrophage-binding probiotic delivery systems enhance retention within inflamed intestinal regions and synergistically suppress inflammation [170]. Beneficial microbiota-derived vesicles further function as interkingdom signaling mediators [171,172]. For example, vesicles derived from *Faecalibacterium prausnitzii* have been shown to alleviate viral intestinal injury and secondary inflammation by reshaping microbial metabolic networks and promoting mucosal repair.

Collectively, biomimetic membrane-based nanodelivery systems for intestinal infections have evolved from antimicrobial carriers into integrated therapeutic platforms. These systems can support toxin decoy neutralization, inflammatory cascade modulation, and microbiome restoration. This transition reflects a broader shift from pathogen-centered intervention toward systems-level regulation of host–pathogen–microbiome interactions. It also suggests an emerging engineering framework for managing complex intestinal infections and infection-associated dysbiosis.

## 5. Challenges and Future Priorities of Cell Membrane-Based Delivery Systems

Although cell membrane–based nanocarriers offer clear advantages in immune evasion, biocompatibility, and biological targeting, their clinical translation remains limited by several unresolved technical and regulatory barriers. Unlike fully synthetic nanomaterials, membrane-based delivery systems are derived from biologically complex structures whose function depends on preservation of native molecular organization. Their performance is therefore highly sensitive to perturbations introduced during membrane isolation, coating assembly, quality control, and scale-up. At a fundamental level, these challenges reflect the fact that biological membranes are not passive materials but dynamic interfaces whose function depends on composition, topology, and interfacial integrity. From a translational perspective, the three highest priorities are: (1) establishing standardizable membrane sources while preserving functional topology; (2) developing quantitative quality-control methods that can distinguish functionally valid membrane coverage from merely apparent coating; and (3) generating long-term biosafety data together with GMP-compatible manufacturing workflows.

### 5.1. The Three Highest Priorities for Clinical Translation

These priorities are closely linked rather than independent. Membrane source variability affects coating quality, coating quality affects biological validity, and both ultimately shape biosafety, manufacturability, and regulatory acceptability. For this reason, future progress will depend less on adding new membrane types than on improving standardization, measurement, and translational robustness.

#### 5.1.1. Membrane Origin & Topological Integrity

The biomimetic functionality of CMNPs depends on the asymmetric organization of membrane proteins, glycans, and lipid bilayers, which collectively govern immune recognition, cellular adhesion, and tissue targeting [16]. For this reason, one of the most immediate translational priorities is to preserve membrane purity, biological activity, and native topological orientation during isolation, especially for membranes derived from nucleated cells. Current extraction strategies typically rely on hypotonic lysis followed by differential or density-gradient centrifugation [173]. Although these approaches can yield usable membrane fractions, they often fail to completely remove organelle debris, nucleic acids, and cytoplasmic residues. Such contaminants can alter surface composition and may trigger unintended immune activation, thereby compromising both biosafety and functional stability. In addition, extrusion and ultrasonic disruption can alter membrane organization and lead to random inversion of transmembrane protein orientation [174,175]. Importantly, membrane functionality depends not only on protein preservation but also on maintenance of native topology and extracellular accessibility [175]. If receptor orientation is disrupted during membrane remodeling, molecular recognition may be impaired even when total protein content appears unchanged, highlighting the limitations of composition-based characterization alone [176]. Future work should therefore move from recovery-driven extraction toward function-preserving membrane preparation. Gentle lysis, hierarchical purification, and standardized evaluation metrics based on functional protein exposure and orientation integrity will be essential if membrane sourcing is to become reproducible and regulatory-compatible.

#### 5.1.2. Quantitative Quality Control of Coating Completeness and Functional Reconstruction

A second major priority is to establish quantitative quality-control methods that can verify whether membrane coating is complete and functionally meaningful rather than merely apparent. Recent mechanistic studies show that membrane coating is not a deterministic encapsulation event but a thermodynamically governed self-assembly process regulated by membrane biophysical properties [175]. During assembly, membrane vesicles adsorb, rupture locally, and reorganize across the nanoparticle surface. Formation of an intact bilayer depends on sufficient membrane fluidity to enable fusion and repair of structural discontinuities. When membrane mobility is limited, partially coated nanoparticles with discontinuous coverage can result. Importantly, such structures may be misinterpreted as fully coated particles if validation relies mainly on TEM morphology [175]. Partial coating can profoundly alter biological performance by exposing nanocore regions and changing immune evasion, protein corona formation, and cellular uptake. Modulation of membrane fluidity, for example by phospholipid supplementation, may improve complete coating efficiency and delivery performance [157]. Successful membrane adsorption should therefore not be equated with functional biomimetic reconstruction. For future translation, coating integrity should be treated as a critical quality attribute (CQA) and assessed by quantitative methods such as fluorescence quenching, protein accessibility mapping, and membrane continuity evaluation. Moving beyond morphology-based validation toward mechanism-informed QC will be essential for predictable and scalable manufacturing [175].

#### 5.1.3. Long-Term Biosafety, Immunogenicity, and GMP-Compatible Scale-Up

A third priority is to demonstrate long-term biosafety and immunological tolerability while developing GMP-compatible scale-up strategies. This is particularly important because membrane-based systems are derived from biological materials whose structural and functional properties are highly sensitive to production variability. During scale-up, variability in membrane source materials remains a major concern. For in vitro-cultured cell sources, prolonged passaging can lead to genetic drift or phenotypic changes, with batch-to-batch consequences for membrane composition and function. Standardized master and working cell banks, together with continuous monitoring of cellular identity and function, are therefore essential. Process-related variability further complicates large-scale production. Critical parameters such as temperature, pH, shear stress, flow dynamics, and ultrasonic energy input can all affect membrane integrity and delivery performances [177]. Incomplete removal of free membrane fragments or uncoated nanoparticles may also alter in vivo safety profiles, making purification a critical manufacturing step. Clinical translation will additionally require sterile processing, endotoxin control, validated filtration, and long-term storage assessment under GMP-compatible conditions. Equally important, future studies should move beyond short-term proof-of-concept safety data and include longer-term immunogenicity and biodistribution assessment, particularly for repeated dosing. Successful scale-up will ultimately depend on linking critical process parameters (CPPs) and CQA in a reproducible framework. Taken together, future translation will require biosafety datasets and manufacturing workflows that are robust enough for regulatory evaluation rather than only laboratory reproducibility.

### 5.2. Additional Priorities for Intestinal Delivery Systems

Beyond these general translational priorities, intestinal delivery introduces several additional barriers that are especially relevant for CMNP-based systems. First, gastrointestinal acidity, digestive enzymes, and bile salts can destabilize membrane architecture and impair protein function before particles reach the intended site [178]. Improving oral stability under gastrointestinal conditions is therefore a major additional priority. Second, the intestinal mucus layer presents a difficult balance rather than a simple barrier. Moderate mucus interaction may support retention, but excessive mucoadhesion promotes clearance, whereas insufficient interaction limits epithelial penetration [179]. Future intestinal systems will therefore need adaptive interfaces that balance penetration with local retention. Third, epithelial translocation remains poorly understood and inefficient. Although microfold (M) cell transport and transcytosis have been proposed, nanoparticle trafficking across enterocytes and Peyer’s patches is still not sufficiently defined. More predictive mechanistic models of epithelial transport are therefore needed [180]. In addition, the gut microbiome is emerging as an underappreciated determinant of CMNPs performance. Microbial activity may degrade membrane structures, reshape protein corona formation, and alter local immune responses, thereby influencing stability and targeting efficiency [180]. This suggests that microbiota-responsive or microbiome-aware delivery design may become increasingly important for intestinal applications [181]. Overall, intestinal applications add a second layer of translational difficulty beyond general CMCT manufacturing and quality-control issues. Progress in this area will depend on combining the three general priorities outlined above with improved oral stability, mucus navigation, epithelial transport understanding, and microbiome-aware design.

## 6. Conclusions

CMCT represents an important development in nanomedicine design, shifting attention from conventional chemically engineered surface functionalization toward membrane-based biological interface engineering. Rather than recreating isolated functions through synthetic modification, CMCT uses cellular membranes to transfer multiple biologically relevant surface features onto synthetic nanomaterials. This design strategy allows synthetic nanoparticles to interact with biological environments through more compatible surface interfaces, thereby expanding the functional scope of nanocarriers in physiological systems.

The resulting CMNPs combine the structural programmability of synthetic cores with the functional advantages of biological membranes. These functionalities include immune evasion, prolonged circulation, lesion homing, and microenvironment responsiveness. In the context of intestinal drug delivery, these biohybrid systems offer a useful approach to overcoming gastrointestinal barriers by combining physicochemical protection with biologically mediated targeting and immune modulation. As discussed in this review, the use of various membrane sources and hybrid engineering strategies has expanded CMNPs from passive delivery vehicles into multifunctional systems that can support inflammation control, toxin neutralization, tumor targeting, and microbiome-associated intervention.

Despite these advances, CMCT remains at an early translational stage. Key challenges, including the preservation of membrane integrity and protein orientation, a mechanistic understanding of coating completeness, scalable manufacturing, and regulatory standardization, must be addressed before broader clinical implementation can be considered. At present, translation is also limited by the lack of human data, the small number of large-animal studies, high regulatory uncertainty, and the absence of widely accepted manufacturing standards. Future progress will therefore depend not only on improving fabrication efficiency, but also on establishing mechanism-informed biomanufacturing frameworks and quantitative quality-control standards that are compatible with GMP requirements.

Looking ahead, CMNPs research is likely to move beyond static biomimicry toward more controllable and responsive biointerface design. The integration of genetic engineering, stimulus-responsive materials, and microbiome-aware designs may support delivery systems that respond more effectively to disease-specific intestinal microenvironments. In this context, future progress may depend on designing delivery systems that work with biological complexity more effectively, rather than relying only on static material-level mimicry.

Overall, CMCT-based technologies provide a useful framework for integrating materials science, immunology, and bioengineering in drug-delivery design. Continued interdisciplinary progress may improve the translational potential of membrane-engineered nanomedicine for both intestinal and systemic diseases, although this will depend on stronger manufacturing control, more rigorous preclinical evaluation, and eventual clinical validation.

## Data Availability

No new data were created or analyzed in this study.
